# Mitotic-Chromosome-Based Physical Mapping of the *Culex quinquefasciatus* Genome

**DOI:** 10.1371/journal.pone.0115737

**Published:** 2015-03-13

**Authors:** Anastasia N. Naumenko, Vladimir A. Timoshevskiy, Nicholas A. Kinney, Alina A. Kokhanenko, Becky S. deBruyn, Diane D. Lovin, Vladimir N. Stegniy, David W. Severson, Igor V. Sharakhov, Maria V. Sharakhova

**Affiliations:** 1 Department of Entomology and Fralin Life Science Institute, Virginia Tech, Blacksburg, Virginia, United States of America; 2 Department of Genomics, Bioinformatics, and Computational Biology, Virginia Tech, Blacksburg, Virginia, United States of America; 3 Institute of Biology and Biophysics, Tomsk State University, Tomsk, Russia; 4 Department of Biological Sciences and Eck Institute for Global Health, University of Notre Dame, Notre Dame, Indiana, United States of America; University of Massachusetts Medical School, UNITED STATES

## Abstract

The genome assembly of southern house mosquito *Cx*. *quinquefasciatus* is represented by a high number of supercontigs with no order or orientation on the chromosomes. Although cytogenetic maps for the polytene chromosomes of this mosquito have been developed, their utilization for the genome mapping remains difficult because of the low number of high-quality spreads in chromosome preparations. Therefore, a simple and robust mitotic-chromosome-based approach for the genome mapping of *Cx*. *quinquefasciatus* still needs to be developed. In this study, we performed physical mapping of 37 genomic supercontigs using fluorescent in situ hybridization on mitotic chromosomes from imaginal discs of 4th instar larvae. The genetic linkage map nomenclature was adopted for the chromosome numbering based on the direct positioning of 58 markers that were previously genetically mapped. The smallest, largest, and intermediate chromosomes were numbered as 1, 2, and 3, respectively. For idiogram development, we analyzed and described in detail the morphology and proportions of the mitotic chromosomes. Chromosomes were subdivided into 19 divisions and 72 bands of four different intensities. These idiograms were used for mapping the genomic supercontigs/genetic markers. We also determined the presence of length polymorphism in the q arm of sex-determining chromosome 1 in *Cx*. *quinquefasciatus* related to the size of ribosomal locus. Our physical mapping and previous genetic linkage mapping resulted in the chromosomal assignment of 13% of the total genome assembly to the chromosome bands. We provided the first detailed description, nomenclature, and idiograms for the mitotic chromosomes of *Cx*. *quinquefasciatus*. Further application of the approach developed in this study will help to improve the quality of the southern house mosquito genome.

## Introduction

Mosquito-borne infectious diseases pose unacceptable risks to public health and welfare [1]. Among mosquitoes, species of the genus *Culex* are the most taxonomically diverse and geographically widespread [2,3]. Mosquitoes within the *Cx*. *pipiens* complex are major vectors for lymphatic filariasis caused by nematode *Wuchereria bancrofti* in tropical and subtropical regions of Asia, Africa, Central and South America, and Pacific Islands. *Cx*. *quinquefasciatus* is also a primary vector for arboviral infections, such as West Nile virus, St. Louis encephalitis, Sindbis, and Rift Valley fever viruses. Members of the *Cx*. *pipiens* complex have great variation in their host range, feeding behavior, and female diapause. Sequencing of the genomes for three major mosquito taxa, *Anopheles gambiae* [4], *Aedes aegypti* [5], and *Cx*. *quinquefasciatus* [6], provides important insights into genetic diversity of mosquitoes and evolution of the mosquito-pathogen interactions [7]. However, compared to other mosquitoes, *Cx*. *quinquefasciatus* has the most fragmented genome. A total of 579 Mb (mega base pairs) is currently assembled into 3,171 supercontigs with the N50 size being ∼476 Kb (kilo base pairs). The N50 supercontig sizes are 12.3 Mb in the *An*. *gambiae* (PEST) genome and 1.5 Mb in the *Ae*. *aegypti* genome. A lack of a high-quality chromosome-based genome assembly for *Cx*. *quinquefasciatus* remains a significant impediment to further progress in *Cx*. *quinquefasciatus* biology and comparative genomics of mosquitoes. Fragmented unmapped genome assemblies create substantial problems for genome analysis. For example, unidentified gaps cause incorrect or incomplete annotation of genomic sequences; unmapped sequences lead to confusion between paralogous genes and genes from different haplotypes, and the lack of chromosome assignment and orientation of the sequencing contigs does not allow for studying chromosome organization and evolution [8]. Therefore, utility of the genome assembly for investigations on basic biology requires anchoring of the genomic supercontigs onto *Cx*. *quinquefasciatus* chromosomes.

Among other approaches, genetic linkage mapping has been so far the most effective method for the genome mapping of *Cx*. *quinquefasciatus*. Among closely related *Cx*. *pipiens* species, several morphological mutants have been described by Leonore Dennhofer [9]. Crosses involving different mutants permitted assignment of genes related to these mutations to three linkage groups. Use of deoxyribonucleic acid (DNA) markers as a new approach allowed the construction of a genetic map which originally consisted of 21 complementary DNA (cDNA) markers and covered 7.1, 80.4, and 78.3 cM on chromosomes 1, 2, and 3, respectively [10]. The sex determination locus was genetically mapped to the smallest linkage group 1 in *Cx*. *pipiens*. In addition, multiple quantitative trait loci (QTL), related to differences in reproductive diapause between species in the *Cx*. *pipiens* complex, were also genetically mapped [11]. The most recent genetic linkage map developed for *Cx*. *quinquefasciatus* includes 63 genetic loci [12]. This map covered 29.5, 88.8, and 65.6 cM on linkage groups 1, 2, and 3 and allowed integration of 10.4% of the genome with the genetic linkage map. Currently, this is the most representative map of the *Cx*. *quinquefasciatus* genome. However, this map has never been integrated with cytogenetic maps developed for this mosquito.

Physical mapping in *Cx*. *quinquefasciatus* is challenging because of the poor quality of the polytene chromosomes. Several attempts to create a cytogenetic photomap using *Cx*. *quinquefasciatus* polytene chromosomes have been made. The Malpighian tubule chromosome map for *Cx*. *pipiens* [13] and *Cx*. *quinquefasciatus* [14] and, more recently, the salivary gland chromosome map for *Cx*. *quinquefasciatus* [15], were developed. However, correspondence of arms and regions among these maps and the original drawn map published by L. Dennhofer [16] is uncertain. Almost no similarities between landmarks of different chromosome maps were found [15]. These problems occurred because of low levels of polyteny, high frequency of ectopic contacts or associations of nonhomologous chromosome regions, and poor spreading of *Cx*. *quinquefasciatus* polytene chromosomes in preparation. As a result, only two genes for esterase- and odorant-binding proteins were mapped to the polytene chromosomes of *Cx*. *quinquefasciatus* [15,17].

In contrast to polytene chromosomes, mitotic chromosomes do not form ectopic contacts and can be easily utilized for mapping DNA probes to the chromosome bands. A simple and robust technique for obtaining high-quality mitotic chromosomes from imaginal discs of 4^th^ instar larvae was recently developed for the yellow fever mosquito *Ae*. *aegypti* [18]. This work resulted in 13%, and more recently, in 45% of the genome placement to the chromosomes for this mosquito [19,20]. Mitotic chromosomes of *Cx*. *pipiens*, the closest relative of *Cx*. *quinquefasciatus*, have been briefly described in 1963 as three pairs of metacentric chromosomes and numbered in order of increasing size as chromosomes I, II, and III [21]. In some cases, chromosome I was identified as a submetacentric chromosome, meaning that the relative length of the shorter arm p was less than 35% of the total chromosome length. It was also determined that *Cx*. *pipiens* chromosomes are smaller than those in *Ae*. *aegypti*. Chromosome measurements also demonstrated that compared with *Ae*. *aegypti* chromosomes *Cx*. *pipiens* chromosome I was disproportionally smaller than chromosomes II and III. Unlike in anophelines that have heteromorphic X and Y sex chromosomes [22], sex-determining chromosomes in *Cx*. *quinquefasciatus* were considered as homomorphic. Only two genes, 18S and 28S ribosomal DNA (rDNA), have been physically mapped to the smallest mitotic chromosome of *Cx*. *pipiens* [23]. Chromosome maps suitable for the physical mapping have not been developed for the mitotic chromosomes of *Cx*. *quinquefasciatus*.

In this study, mitotic chromosomes of *Cx*. *quinquefasciatus* were described in details and directly linked to the previously established genetic linkage groups by hybridization of 26 Bacterial Artificial Chromosome (BAC) probes associated with 58 genetic markers [24] to the chromosomes. As a result, chromosomes were renumbered according to the existing genetic linkage groups [10,12]. We also developed idiograms or schematic representation of chromosome banding patterns for *Cx*. *quinquefasciatus*. In addition, we mapped an 18S rDNA probe and 9 large genomic supercontigs to the chromosomes. Thus, our study has demonstrated that a mitotic chromosome band-based technique can be utilized for further development a high-resolution physical genome map for the *Cx*. *quinquefasciatus*.

## Materials and Methods

### Mosquito strain and slide preparation

The laboratory strain Johannesburg (JHB), used in this study was obtained from BEI Resources [25]. This colony was originated from the field population of *Cx*. *quinquefasciatus* near Johannesburg, South Africa [15]. The same strain was previously used for the genome sequencing project [6]. Adult mosquitoes were kept at 26°C and fed on artificial membrane blood feeders 4–5 days after emerging. Approximately 4 days after feeding, the eggs were collected and hatched at 26°C. After 4 days, 2^nd^ instar larvae were transferred to 16°C to obtain a high number of mitotic divisions in imaginal discs [18]. At 7–8 days, 4^th^ instar larvae were used for slide preparation. Our study utilized mitotic chromosomes from imaginal discs of 4^th^ instar larvae which develop into legs and wings at the adult stage. These imaginal discs are located immediately under the cuticle and can be easily dissected from the larvae. The morphology of the imaginal discs and details of their dissection from the larvae were previously described for three species of mosquitoes including *Cx*. *quinquefasciatus* [26]. Chromosome preparations were made using a routine technique based on hypotonic treatment and subsequent application of Carnoy’s solution (3 parts of ethanol: 1 part of acetic acid) and 50% propionic acid [26]. The percentage of chromosome preparations suitable for further analysis, which contained more than 50 chromosome spreads, was ∼85%.

### DNA probe preparation and fluorescent *in situ* hybridization (FISH)

Notre Dame Johannesburg (NDJ) BAC library [24] was used as a probe DNA source for FISH. BAC clone DNA isolation and sequencing were performed at the Clemson University Genomics Institute. BAC clone correspondence to the certain genomic supercontigs or genetic markers was determined by BAC library screening [26] or by BAC-end sequence comparison using Basic Local Alignment Search Tool (BLAST) against the genome assembly of *Cx*. *quinquefasciatus* available at Vectorbase [27]. Three polymerase chain reaction (PCR) fragments with sizes ∼1Kb from genomic supercontig 3.32 were amplified using primers: AAAACCCATCTCCCTCGTAG forward, GCTTCTCCAAAACCTTCCTC reverse; TCAAACGACCACAACTTTGA forward, TGGCCTTGTTCTTCTTCTTG reverse; and ATGAAGTTACGGTCGTCAGC forward, AGTGCATGATGACTCCCATT reverse. Probes were labeled by nick translation with Cy3- or Cy5-deoxyuridine 5-triphosphate (dUTPs) (GE Healthcare UK Ltd., Buckinghamshire, UK) as described before [26]. An 18S rDNA probe was amplified using forward primer CCTATATGGTGGCGCTTGAT and reverse primer AACTAAGAACGGCCATGCAC. It was labeled by Cy3- or Cy5-dUTPs in a PCR reaction using PCR IMMOMIX (Bioline USA, Taunton, MA) with standard parameters. Nonspecific hybridization of BAC DNA probes to the chromosomes was prevented by pre-hybridization of the probe with unlabeled repetitive DNA fractions of genomic DNA [26]. Genomic DNA was extracted from adult mosquitoes using Qiagen Blood & Cell Culture DNA Maxi Kit (Qiagen Science, Germantown, MD, USA). Approximately 500 mg of adult mosquitoes were taken for extraction. Final outcome of repetitive DNA fractions accounts for ∼20% of genomic DNA. Approximately 250–350 ng of DNA probe were pre-hybridized with 4 mg of repetitive DNA. FISH of DNA probes was performed using a standard protocol [26]. Slides were analyzed using Zeiss LSM 510 Laser Scanning Microscope (Carl Zeiss Microimaging, Inc., Thornwood, NY, USA) at 600X magnification. For each probe, from 5–10 chromosome spreads were tested.

### Image processing and measurements

For idiogram development, the best images of the chromosomes from imaginal discs stained with Oxasole Yellow (YOYO-1) iodide (Invitrogen Corporation, Carlsbad, CA, USA) were selected. The original images were converted into gray-scale images and contrasted as described previously [28]. These chromosome images were straightened and aligned for comparison using ImageJ program [18,29]. In total, 150 chromosomes at early metaphase were analyzed, and 25–30 images of each chromosome with reproducible banding patterns were used for idiogram development. To calculate exact proportions of chromosomes, we utilized standard curve measurements in Zen2009LightEdition software [30]. We utilized early metaphase and mid-metaphase chromosomes to precisely assign signals to the particular chromosome band.

## Results

### *Culex quinquefasciatus* chromosome nomenclature

According to an original chromosome nomenclature, three pairs of metacentric chromosomes of *Cx*. *pipiens*, the closest relative of *Cx*. *quinquefasciatus*, were numbered as I, II, and III in order of increasing size (Rai, 1963). In this study, we established correspondence between mitotic chromosomes and genetic linkage groups by direct placement of 26 genomic supercontigs associated with 58 genetic markers to the chromosomes (Table 1). These markers were previously mapped to smallest, largest, and intermediate linkage groups 1, 2, and 3 of *Cx*. *pipiens*, respectively [10]. Seven BAC clones for this mapping were identified by screening the NDJ BAC library [24] using PCR-amplified genetic markers [31]. Another 19 BAC clones were identified as belonging to the genomic supercontigs with known genetic markers by BAC-end sequencing followed by BLAST-based alignment to the genomic sequences of *Cx*. *quinquefasciatus* [27]. BAC clones corresponding to linkage group 1 carrying markers CX60, LF284, and 8 microsatellite markers were mapped to the smallest chromosome. Markers consisting of 6 complementary DNA (cDNA) and 16 microsatellites from linkage group 2 were mapped to the largest chromosome. Seven cDNA and 17 microsatellite markers from linkage group 3 were mapped to the intermediate-in-size chromosome. Supercontig 3.32 containing genetic marker LF335 was mapped as 3 PCR-amplified products with sizes ∼1 Kb. The order of most markers on chromosomes exactly followed their positions in the genetic linkage map. We found only two discrepancies in the order of markers CX44 and LF203 on chromosome 2 and also a BAC clone with genetic marker LF108 was mapped on a different chromosome. Thus, we propose renumbering the mitotic chromosomes for *Cx*. *quinquefasciatus* in correspondence to the genetic linkage groups as follows: 1—smallest, 2—largest, and 3—intermediate chromosomes.

**Table 1:** List of genomic supercontigs, BAC clones, and genetic markers mapped to the chromosome of *Cx*. *quinquefasciatus*.

SC	SC size	BAC well/plate	AC# (T7)	AC# (M13)	Genetic marker (AC#)	Location
3.845	193967	NDJ.020P4	GF110931	N/A	N/A	1p12
3.14	1835525	NDJ.001J24	KG777556	N/A	N/A	1p21
3.36	1496554	NDJ.003F11	KG961588	KG961589	C36GTT1	1p31
3.12	1895535	NDJ.003N20	KG961610	KG961611	C12CCT1, C12GTT1, C12GTC, Cxpq51	1p33
3.56	1226699	NDJ.001O10	KG777559	KG777560	LF284 (BM005502), C56GCA1, C56TGT1	1q12
3.134	821072	NDJ.001D17	KG777561	KG777562	C134AC1b	1q12
3.1464	65637	N/A	N/A	N/A	18S rDNA	1q13
3.49	1394590	NDJ.002A07	KG961590	KG961591	N/A	1q21
3.127	851360	NDJ.048G24	N/A	N/A	CX60 (FD664718)	1q31
3.129	873558	NDJ.064F11	N/A	N/A	CX107 (FD664723), C129CT1	2p25
3.280	510693	NDJ.001J13	KG961592	KG961593	C28GT1	2p31
3.66	1106043	NDJ.002N10	KG961586	KG961587	C66CGT1, C66GT1, C66TGT1	2p32
3.15	1741670	NDJ.002C09	KG777563	KG777564	N/A	2p34
3.177	728683	NDJ.001N13	KG961594	KG961595	CX114 (FD664728), C177CGT1	2p34
3.186	747982	NDJ.001K16	KG961596	KG961597	C186TGT1	2p35
3.5	2487969	NDJ.002C07	KG777565	KG777566	CX44 (FD664710), C5CGT1, C5GTG1	2q11
3.11	2034973	NDJ.001N24	KG777567	KG777568	N/A	2q12
3.9	2056888	NDJ.001E14	KG777571	KG777572	N/A	2q13
3.17	1689851	NDJ.001E04	KG777569	KG777570	N/A	2q17
3.95	956384	NDJ.003F24	KG961598	KG961599	LF203 (BM005503), C95CAG1, C95GCA1	2q22
3.4	2511003	NDJ.002L02	KG961600	KG961601	C4TTG1	2q25
3.18	1726395	NDJ.001D20	KG777573	KG777574	N/A	2q32
3.10	2129711	NDJ.001J08	KG777575	KG777576	N/A	2q32
3.68	1113402	N/A	N/A	N/A	LF335 (BM005505), C68TCG1	2q42
3.13	1876709	NDJ.003H21	KG961602	KG961603	C13TC1	2q43
3.32	1521851	NDJ.033G10	N/A	N/A	LF334 (BM005506), C32AC1, C32AG1, C32TC1b, C32TGC1	2q43
3.65	1116611	NDJ.005K08	N/A	KG961581	C65AC1, C65CAG1	3p11
3.446	375653	NDJ.009F06	N/A	N/A	CX11 (FD664697), C446TC1	3p32
3.208	649753	NDJ.003E21	N/A	N/A	C208GCA1, CxqTri4	3p32
3.1	3873040	NDJ.001G13	KG777581	KG777582	C1CAG1	3q12
3.67	1097170	NDJ.002F04	N/A	N/A	LF108 (T58322), C67CT1	3q12
3.73	1095011	NDJ.004O20	KG961605	KG961605	LF272 (BM005484), C73CA1, C73TCG1	3q14
3.139	823831	NDJ.013B09	N/A	N/A	CX53 (FD664714), C139CT1, C139GA1	3q21
3.205	667856	NDJ.005A07	KG961612	KG961613	CX17 (FD664699), C205GAC1, C205GTC1	3q21
3.99	949261	NDJ.024B18	N/A	N/A	CX112 (FD664727), C99GTC1	3q24
3.2	2744360	NDJ.002F15	KG961606	KG961607	C2ACG1	3q32
3.3	2758190	NDJ.001M21	KG777583	KG777584	LF115 (R67978), C3GAC1, C3TGC1	3q33

N/A—not applicable

**Fig 1:**
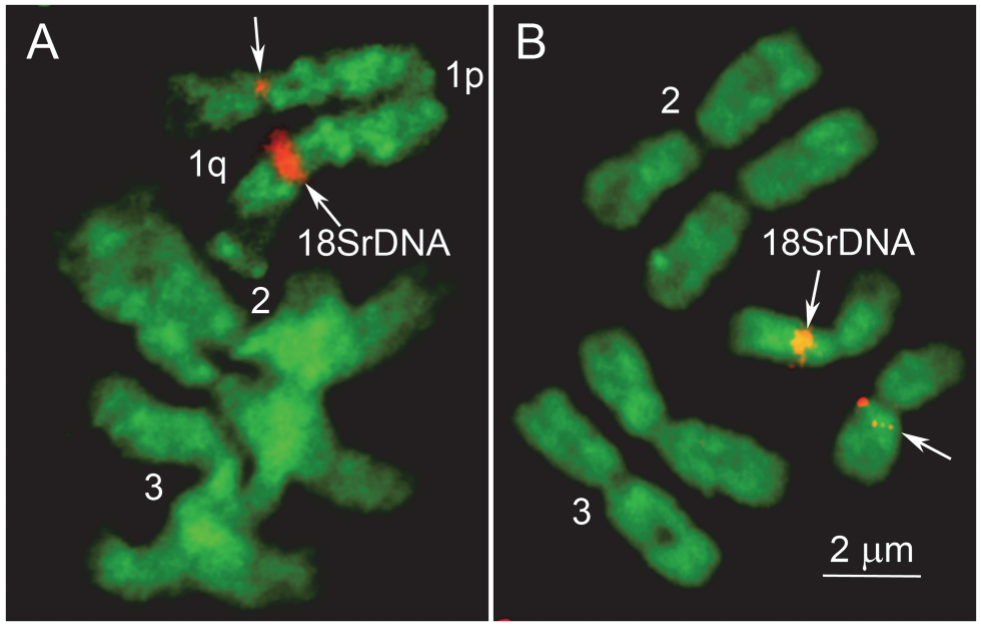
Two variants of sex-determining chromosome 1 at early- (A) and mid-metaphase (B). Position of 18S rDNA probe on chromosome 1 is indicated by arrow.

We also determined the average chromosome lengths at mid-metaphase as 4.04 μm for chromosome 1, 6.37 μm for chromosome 2, and 5.59 μm for chromosome 3 (Table 2). Relative lengths of chromosomes were 25.3%, 39.8%, and 34.9% for each chromosome, respectively. Centromeric indexes (the relative length of the p-arm) were 47.4% and 46.9% for chromosomes 2 and 3. Thus our data confirmed that these two chromosomes are metacentric [21]. Measurements for the centromere position in chromosome 1 varied depending on size of the ribosomal locus determined by FISH of 18S rDNA probe on the chromosome between 43.1% or 48.1%, respectively (Fig. 1). Thus, both variants of chromosome 1 must be also considered as metacentric according to the modern chromosome nomenclature [32].

**Table 2:** The measurements of *Cx*. *quinquefasciatus* mitotic chromosomes from imaginal discs in comparison to *Ae*. *aegypti*.

Mosquito species	*Cx*. *quinquefasciatus*	*Ae*. *aegypti*
**Chromosome 1** Average length, μm	4.04	7.1
Relative length, %	25.30%	28.6%
Centromeric index, %	43.1% or 48.1%	46.9%
**Chromosome 2** Average length, μm	6.37	9.5
Relative length, %	39.80%	37.9%
Centromeric index, %	47.4%	48.6%
**Chromosome 3** Average length, μm	5.59	8.4
Relative length, %	34.9%	33.5%
Centromeric index, %	46.9%	47.4%

### Idiograms of mitotic chromosomes for *Culex quinquefasciatus*

**Fig 2:**
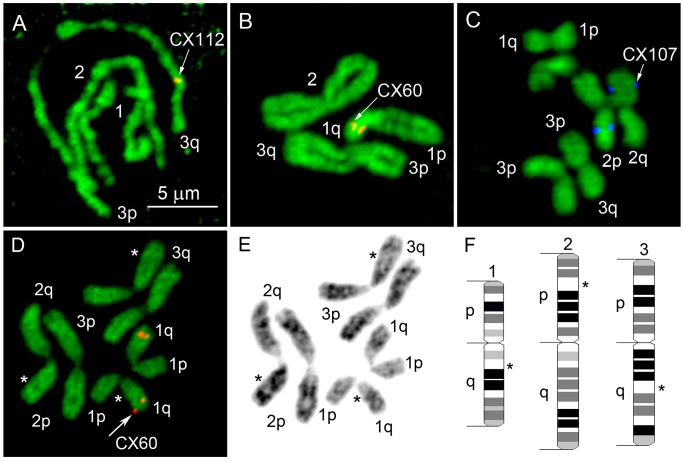
Stages of mitosis (A-C) and chromosome idiogram development (D-F) in *Cx*. *quinquefasciatus*. Early metaphase chromosomes (D) were chosen from prophase (A), prometaphase (B), and late-metaphase (C) chromosomes for the ideogram development. Chromosome images stained with YOYO-1 iodide were converted into gray images (E). Chromosomes on idiograms were subdivided into 72 bands with 4 different intensities (F). Arrows show chromosome positions of the genetic markers CX60 (B, D), CX 112 (A), and CX107 (C). Chromosome landmarks are indicated by asterisks.

In addition to chromosome nomenclature, our study developed idiograms or drawn schematic representations of the banding pattern for mitotic chromosomes of *Cx*. *quinquefasciatus* at early-metaphase. From a whole range of the different stages of mitosis (prophase, prometaphase, metaphase, and anaphase), metaphase chromosomes have the most clear and reproducible banding patterns (Fig. 2). Similarly to *Ae*. *aegypti*, in *Cx*. *quinquefasciatus* homologous chromosomes are paired at prophase and prometaphase (Fig. 2A, B). At these two stages, the visible chromosome number equals three. Chromosomes start segregating from each other at prometaphase and become completely segregated at metaphase (Fig. 2C). Visible chromosome number at metaphase equals six.

For the idiogram development, we used images of the chromosomes at early metaphase stained with YOYO-1 iodide (Fig. 2D). This dye strongly stains euchromatin and therefore provides more detailed banding patterns than DAPI (4',6-diamidino-2-phenylindole), which preferentially stains AT-rich heterochromatic regions [18,26]. The original chromosome pictures were converted into gray-scale images. Chromosome images were then straightened and aligned for comparison. After that, unique and reproducible patterns for each chromosome were identified. Following human chromosome nomenclature [33], we determined four color intensities of the chromosome bands: intense (black), medium intensity (dark gray), low intensity (light gray), and negative (white). Chromosomes were subdivided into 20, 28, and 24 bands for chromosomes 1, 2, and 3, respectively (Fig. 2E). The total number of bands for all chromosomes of *Cx*.*quinquefasciatus* equals 72. Each chromosome of *Cx*. *quinquefasciatus* has unique features or landmarks for the arm recognition: large negative band containing ribosomal locus in the q arm region of chromosome 1, negative band separating intense and medium-intense bands in the p arm of chromosome 2, and large negative band in the middle of arm q on chromosome 3 (Fig. 2).

### Physical mapping on mitotic chromosomes of *Culex quinquefasciatus*

**Fig 3:**
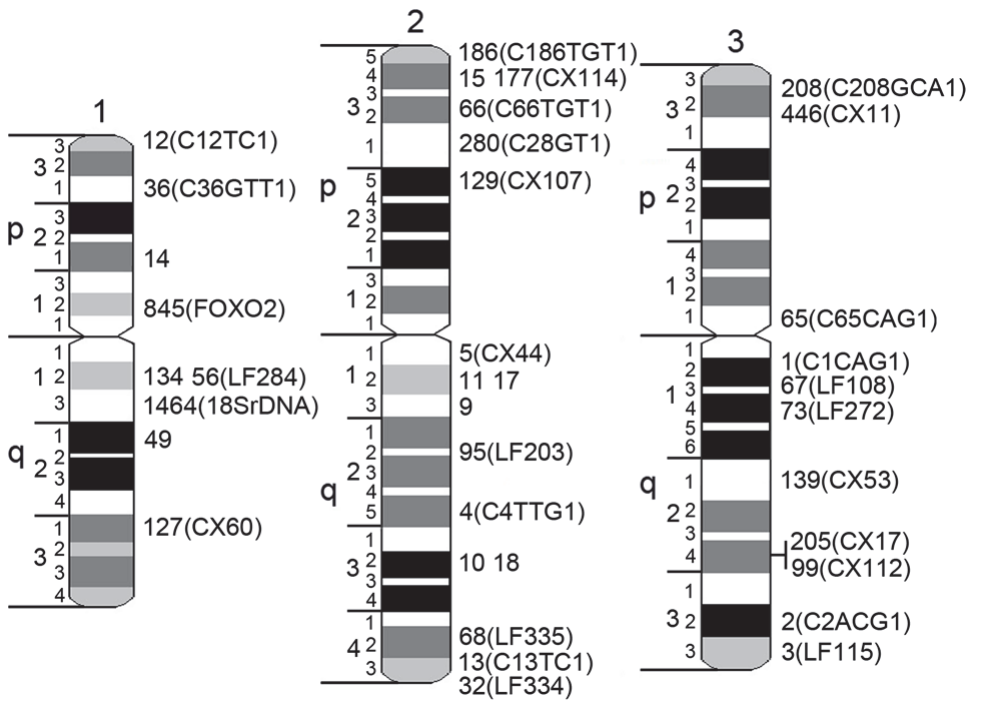
A landmark-guided two-step physical mapping approach on *Cx*. *quinquefasciatus* chromosomes. FISH of two BAC clones of interest was performed in the presence of 2 additional BAC clones, and 18S rDNA used as landmarks for the chromosome arm identification (A-C). Positions of molecular landmarks and 2 BAC clones of interest are indicated by arrows. Mitotic chromosomes at metaphase were used for the rapid assignment of the genomic supercontigs to the chromosome bands (D). Longer prophase (E) or polytene chromosomes (F) were further utilized for ordering the genomic supercontigs within the band.

**Fig 4:**
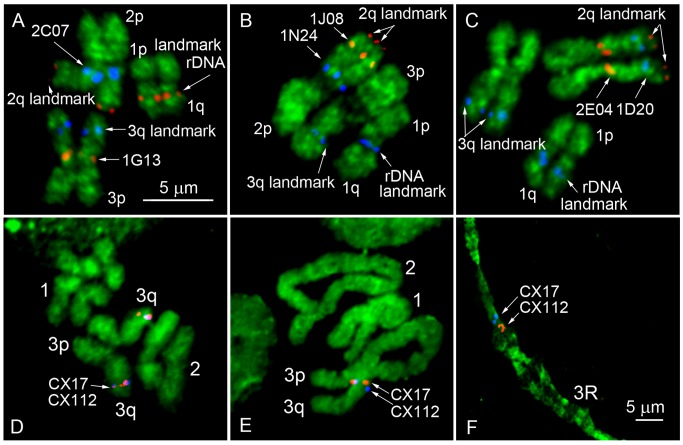
Chromosome idiograms with positions of supercontigs and genetic markers. Chromosomes 1, 2, and 3 are indicated by numbers. Short and long chromosome arms are indicated by letters p and q, respectively. Chromosomes are subdivided into 19 divisions and 72 bands. Genomic supercontigs are indicated by the last 1 to 4 digits of their accession numbers. Genetic markers are shown in brackets.

In addition to BAC clones associated with genetic markers, 9 BAC clones from the largest genomic supercontigs and 18S ribosomal DNA (Table 2) were also mapped to the bands on idiograms by FISH (Fig. 3). An 18S ribosomal DNA probe hybridized above the 2 dark bands on the q arm of chromosome 1 in region 1q13 on the idiogram. In total, a majority of the DNA probes (17) hybridized to the largest chromosome 2, 9 BAC clones were found in intermediate-sized chromosome 3, and 11 DNA probes hybridized to the smallest sex-determining chromosome 1. To simplify physical mapping, we optimized a landmark-guided approach developed for *Ae*. *aegypti* [20,34] for *Cx*. *quinquefasciatus* chromosomes (Fig. 4). We hybridized two BAC clones of interest in the presence of 3 landmark probes: 18S rDNA for 1q arm, telomere BAC clone with genetic marker LF334 on 2q arm, and a BAC clone with genetic marker CX112 close to telomere for 3q arm. Two BAC clones on 3q arm carrying genetic markers CX17 and CX112 were ordered within the band on 3q arm using a two-step mapping approach [19]. In addition to FISH on metaphase mitotic chromosomes (Fig. 4D), the FISH results on prophase and polytene chromosomes were also analyzed. This additional step permitted the ordering of these genetic markers within chromosome band (Fig. 4E, F).

## Discussion

Knowledge about cytogentics of mosquitoes is important to better understand their genome organization and function. However, mitotic chromosomes for the major vector of lymphatic filariasis *Cx*. *quinquefasciatus* were only briefly mentioned, as three pairs of metacentric chromosomes, more than 50 years ago in a review on mosquito mitotic chromosomes [21]. For other mosquitoes such as *An*. *gambiae* and *Ae*. *aegypti*, chromosome work was performed back in the 1970s and 1980s, but it was not done for the *Cx*. *quinquefasciatus*. Here, for the first time, we described the details of morphology, length, and proportions for the mitotic chromosomes of *Cx*. *quinquefasciatus*. Our measurements demonstrated that a total chromosome length at mid-metaphase in *Cx*. *quinquefasciatus* is 1.5 times longer than in *An*. *gambiae* and 1.5 times shorter than in *Ae*. *aegypti* (Table 2) [26]. It reflects the difference in genome sizes of 264 Mb, 579 Mb, and 1376 Mb in *An*. *gambiae*, *Cx*. *quinquefasciatus*, and *Ae*. *aegypti*, respectively. Our measurements also indicated the presence of two variants of sex-determining chromosome 1 in *Cx*. *quinquefasciatus* that differ from each other by the size of ribosomal locus and the centromere position on this chromosome. This polymorphism can be potentially related to the sex determination in this mosquito.

Our study also, for the first time, integrated the cytogenetic map with the genetic linkage map [10,12] and created a new chromosome nomenclature for *Cx*. *quinquefasciatus*. Originally numbered as I, II, and III in order of increasing size [21], chromosomes were renumbered as 1—smallest, 2—largest, and 3—intermediate chromosomes in correspondence to the genetic linkage map of *Cx*. *quinquefasciatus*. The correspondence between genetic linkage groups and chromosomes was determined by direct positioning of 58 genetic markers on the chromosomes. The order of the markers on the chromosomes were basically the same as on the previously published genetic linkage map [12] with only two exceptions in order of the markers CX44 and LF203 on chromosome 2 and position of the genetic marker LF108 on a different chromosome. For *Ae*. *aegypti*, linkage groups were associated with chromosomes based on the analysis of X-ray-generated chromosome translocations in 1970 [35]. The smallest, largest, and intermediate chromosomes were also renumbered as 1, 2, and 3, according to the genetic linkage groups. Recent physical mapping of 100 genetic markers of *Ae*. *aegypti* helped to clarify the chromosome positions of 12 QTL related to pathogen transmission: the filarioid nematode [36], the avian malaria parasite [37,38], and dengue virus [39,40]. These markers were combined into five major clusters of QTL on the chromosome map suggesting that transmission of various pathogens can be controlled by the same genomic loci [19]. However, QTL related to the pathogen transmission for *Cx*. *quinqufasciatus* have not been identified yet. Only multiple QTL to diapause were described [11]. Thus, further development of integrated genetic linkage and chromosome map requires both physical mapping and additional QTL identification.

In addition to chromosome nomenclature, our study developed detailed idiograms for the *Cx*. *quinquefasciatus* chromosomes. The whole chromosome complement was subdivided into 19 regions and 72 bands with four different intensities. We demonstrated the utility of our idiograms for the physical genome mapping by placement of 37 genomic supercontigs to the chromosome locations. Chromosome idiograms for *Cx*. *quinquefasciatus* are comparable to that previously developed for the yellow fever mosquito *Ae*. *aegypti* [18]. The total number of bands (72) is slightly lower in *Cx*. *quinquefasciatus* than in *Ae*. *aegypti* (94). Physical mapping approach based on idiograms allowed assignment of 45% of the *Ae*. *aegypti* genome to chromosome bands [20]. Additional physical mapping, based on the idiograms developed by this study for *Cx*. *quinquefasciatus*, needs to be conducted to increase the genome assignment to the chromosome position.

Using mitotic chromosomes for physical genome mapping raises a concern about the low resolution of this mapping approach compared to traditionally used polytene chromosomes [41–44]. Unlike the subfamily Anophelinae, which have well-developed polytene chromosomes, mosquitoes from the Culicinae subfamily lack high-quality polytene chromosome spreads [14,45]. Nevertheless, our study determined that in addition to mitotic chromosomes low-polytenized chromosomes from salivary glands can be used for the ordering of closely located supercontigs of *Cx*. *quinquefasciatus* without assigning them to specific bands in polytene chromosomes (Fig. 2). This so called “two-step” mapping approach was successfully used for the ordering of 100 genomic supercontigs in *Ae*. *aegypti* [19]. This strategy significantly increased the final resolution of the physical map. The distance between two signals that can be distinguished from each other was estimated at 300 Kb for the polytene chromosomes of *Ae*. *aegypti*. The resolution of polytene chromosomes in *Cx*. *quinquefasciatus* is higher due to their better polytenization and comparable to the 100-Kb resolution of polytene chromosomes in *An*. *gambiae* [42].

Previous investigations of chromosome arm homology between *Cx*. *quinquefasciatus*, *An*. *gambiae*, and *Ae*. *aegypti* indicated whole-arm conservation between *Cx*. *quinquefasciatus* and *An*. *gambiae*, and a whole-arm translocation between chromosomes 2 and 3 of *Cx*. *quinquefasciatus* and *Ae*. *aegypti* [6]. This conclusion was based only on 9%, 31%, and 88% genome placement to the chromosomes for *Cx*. *quinquefasciatus*, *Ae*. *aegypti*, and *An*. *gambiae*, respectively [10,42,46]. The dramatic gene order reshuffling between homologous chromosomes of *Ae*. *aegypti* and *An*. *gambiae* was recently demonstrated based on 45% and 88% genome placement to the chromosomes for these two mosquitoes, respectively [20]. Additional physical mapping may provide some new insights into chromosome evolution in *Cx*. *quinquefasciatus*. For example, FISH results for 18S rDNA suggest an inverted position of the ribosomal locus in chromosome 1 of *Cx*. *quinquefasciatus* compared with *Ae*. *aegypti* [19]. This locus was mapped close to the centromere above the dark bands in *Cx*. *quinquefasciatus* (Fig. 1) but in the middle of the 1q arm below the dark band in *Ae*. *aegypti*. Our measurement data of *Cx*. *quinquefasciatus* chromosomes support the previous observation that the proportions between sex-determining chromosome 1 and autosomes 2 and 3 differ in *Cx*. *pipiens* and *Ae*. *aegypti* (Table 2). It is clear that the relative length of chromosome 1 is shorter in *Cx*. *quinquefasciatus* than in *Ae*. *aegypti*. These results support the idea of partial degradation of the sex-determining chromosome 1 in *Cx*. *quinquefasciatus* compared to chromosome 1 in *Ae*. *aegypti*. Degradation of sex chromosomes was described in different lineages of Drosophila [47]. However, a more advanced chromosome-based genome map for *Cx*. *quinquefasciatus* is required for clarifying the intimate details of chromosome evolution in mosquitoes.

## Conclusion

Our study developed a mitotic-chromosome-based approach for physical mapping of the *Cx*. *quinquefasciatus* genome. We provided the first detailed description and offered a new nomenclature for mitotic chromosomes of *Cx*. *quinquefasciatus*. Based on the genetic linkage map, the smallest, largest, and intermediate chromosomes were numbered as 1, 2, and 3, respectively. We demonstrated the efficiency of our physical mapping approach by placing 37 genomic supercontigs and 58 genetic markers onto chromosome idiograms. This effort, together with previously conducted linkage mapping [12], resulted in the chromosome assignment of 13% of the total genome assembly. Further application of the approach described here will improve the current highly fragmented genome assembly of *Cx*. *quinquefasciatus* and will also stimulate research in vector biology and comparative genomics in mosquitoes.
