# Correction: Mitotic-Chromosome-Based Physical Mapping of the *Culex quinquefasciatus* Genome

**DOI:** 10.1371/journal.pone.0127565

**Published:** 2015-05-08

**Authors:** Anastasia N. Naumenko, Vladimir A. Timoshevskiy, Nicholas A. Kinney, Alina A. Kokhanenko, Becky S. deBruyn, Diane D. Lovin, Vladimir N. Stegniy, David W. Severson, Igor V. Sharakhov, Maria V. Sharakhova

There is an error in the legend for Fig 3, “A landmark-guided two-step physical mapping approach on *Cx*. *quinquefasciatus* chromosomes.” The complete, correct Fig 3 legend is given below.

**Fig 3:**
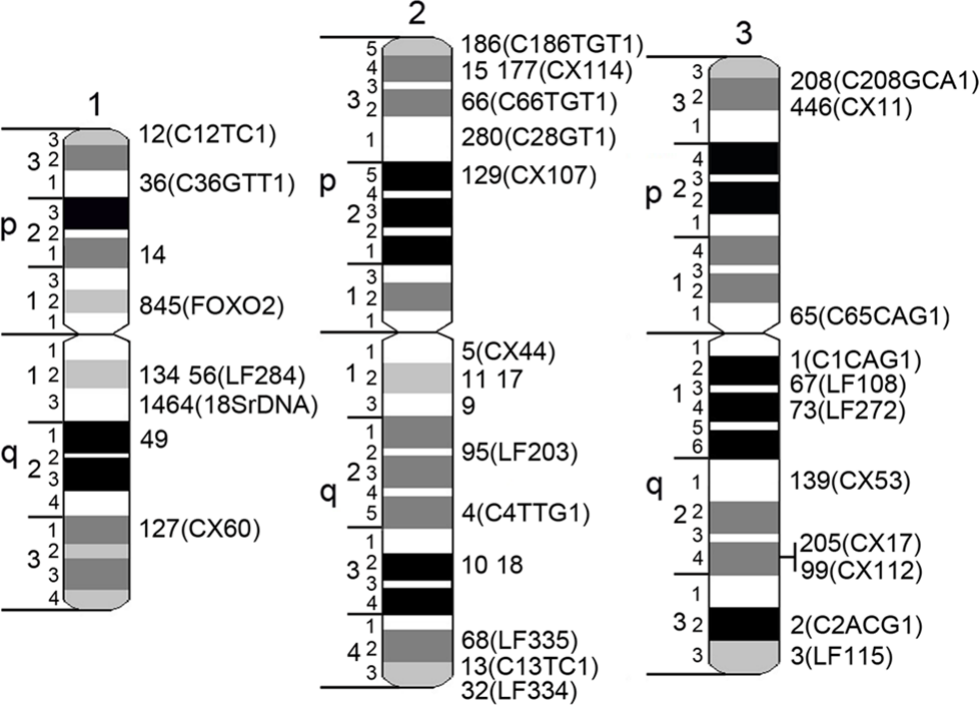
A landmark-guided two-step physical mapping approach on *Cx*. *quinquefasciatus* chromosomes. Chromosomes 1, 2, and 3 are indicated by numbers. Short and long chromosome arms are indicated by letters p and q, respectively. Chromosomes are subdivided into 19 divisions and 72 bands. Genomic supercontigs are indicated by the last 1 to 4 digits of their accession numbers. Genetic markers are shown in brackets.

There is an error in the legend for Fig 4, “Chromosome idiograms with positions of supercontigs and genetic markers.” The complete, correct Fig 4 legend is given below.

**Fig 4:**
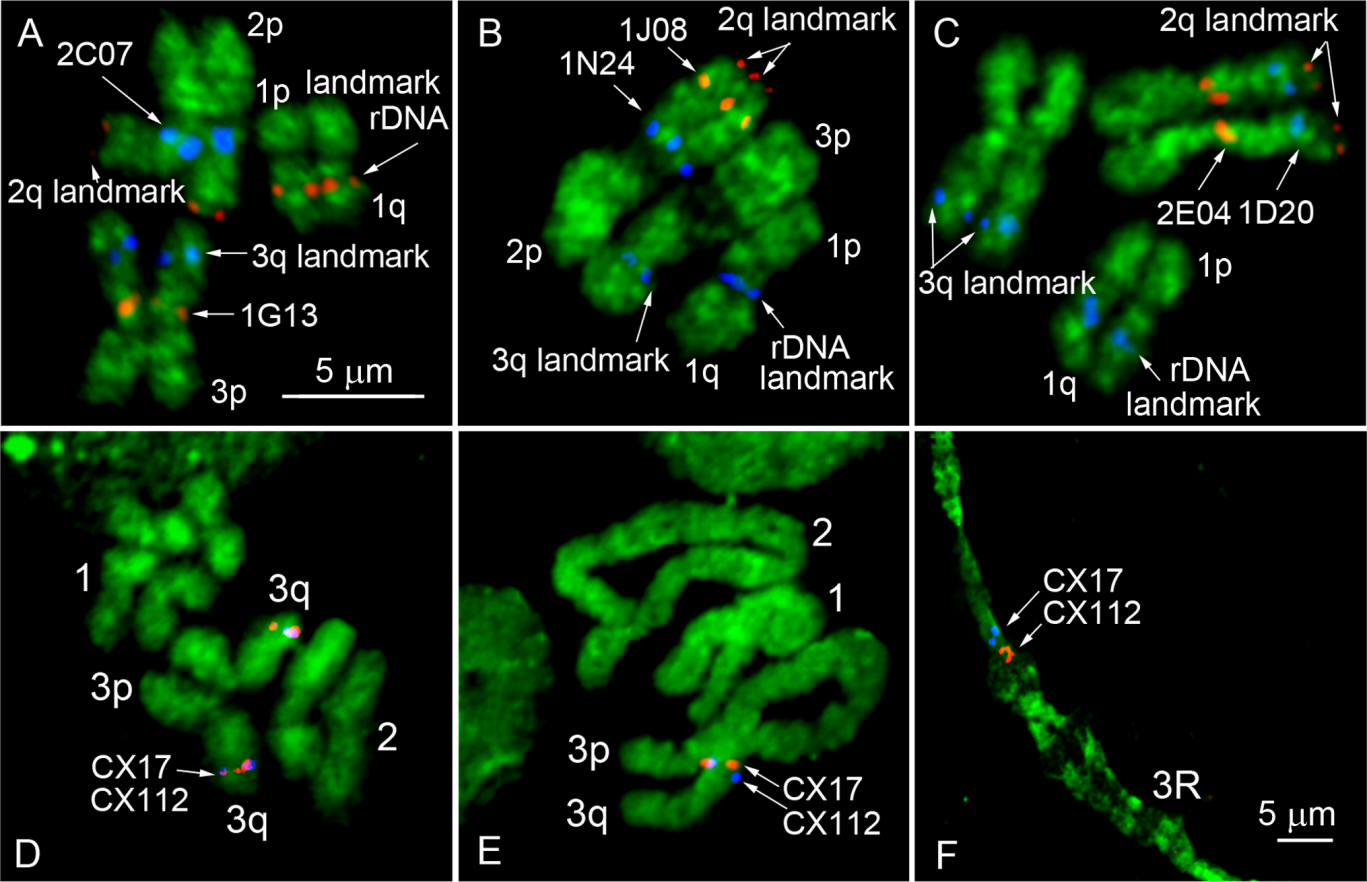
Chromosome idiograms with positions of supercontigs and genetic markers. FISH of two BAC clones of interest was performed in the presence of 2 additional BAC clones, and 18S rDNA used as landmarks for the chromosome arm identification (A-C). Positions of molecular landmarks and 2 BAC clones of interest are indicated by arrows. Mitotic chromosomes at metaphase were used for the rapid assignment of the genomic supercontigs to the chromosome bands (D). Longer prophase (E) or polytene chromosomes (F) were further utilized for ordering the genomic supercontigs within the band.
