# Correction: Correction: Mitotic-Chromosome-Based Physical Mapping of the *Culex quinquefasciatus* Genome

**DOI:** 10.1371/journal.pone.0130108

**Published:** 2015-06-04

**Authors:** The PLOS ONE Staff

There is an error in the Correction published on May 8, 2015. The publisher apologizes for the error. The correct figure legends are:

**Fig 3:**
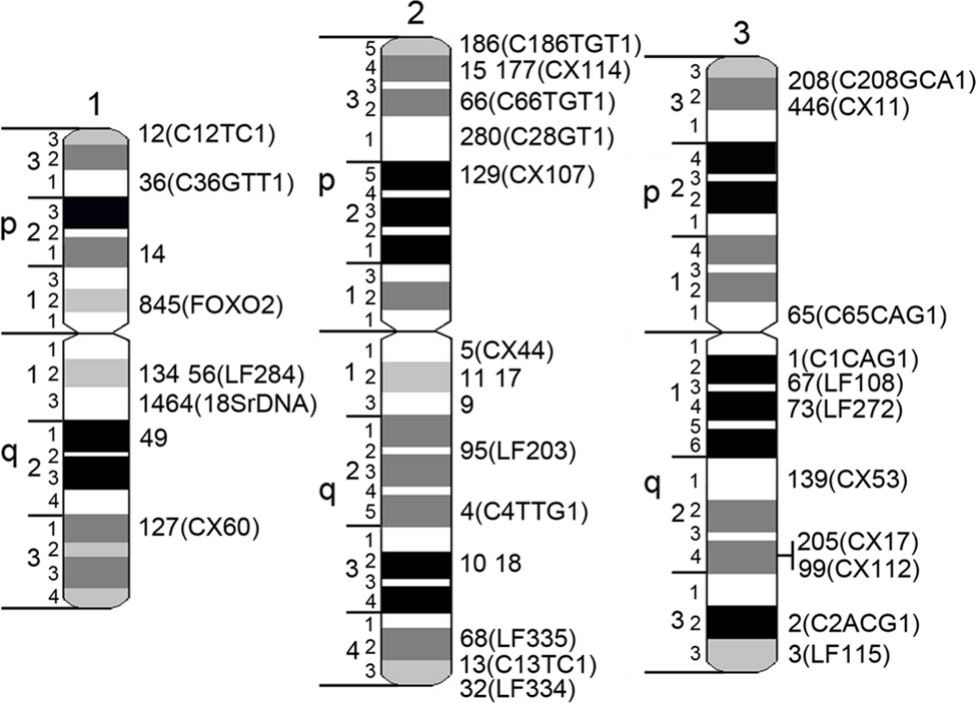
Chromosome idiograms with positions of supercontigs and genetic markers. Chromosomes 1, 2, and 3 are indicated by numbers. Short and long chromosome arms are indicated by letters p and q, respectively. Chromosomes are subdivided into 19 divisions and 72 bands. Genomic supercontigs are indicated by the last 1 to 4 digits of their accession numbers. Genetic markers are shown in brackets.

**Fig 4:**
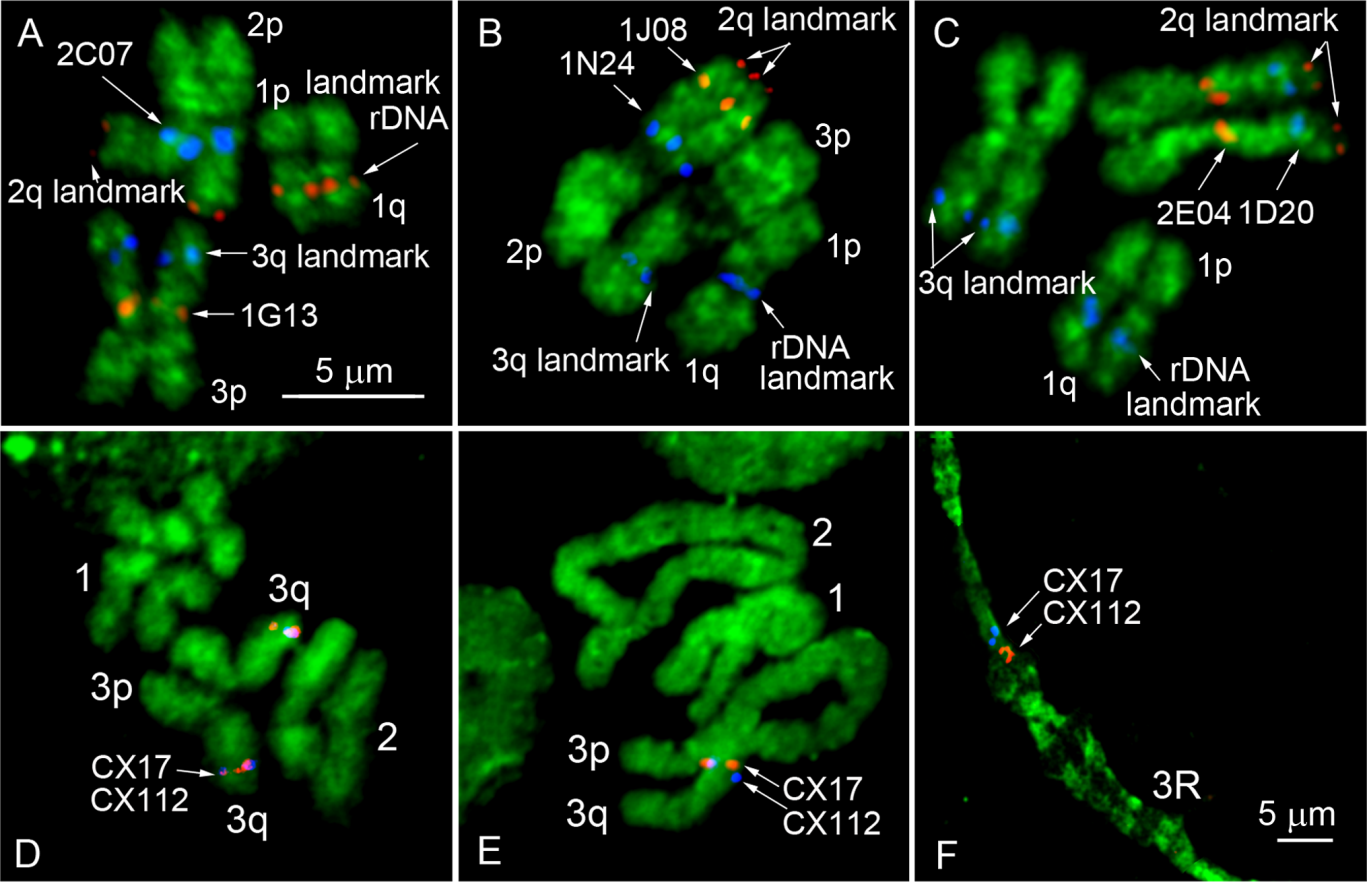
A landmark-guided two-step physical mapping approach on *Cx*. *quinquefasciatus* chromosomes. FISH of two BAC clones of interest was performed in the presence of 2 additional BAC clones, and 18S rDNA used as landmarks for the chromosome arm identification (A-C). Positions of molecular landmarks and 2 BAC clones of interest are indicated by arrows. Mitotic chromosomes at metaphase were used for the rapid assignment of the genomic supercontigs to the chromosome bands (D). Longer prophase (E) or polytene chromosomes (F) were further utilized for ordering the genomic supercontigs within the band.
